# Hospital management of severe bronchopulmonary dysplasia

**DOI:** 10.1186/1824-7288-41-S1-A23

**Published:** 2015-09-24

**Authors:** Corrado Moretti, Caterina S Barbàra, Rosanna Grossi, Stefano Luciani, Paola Papoff

**Affiliations:** 1 Pediatric Emergency and Intensive Care, Department of Pediatrics, Policlinico “Umberto I”, Sapienza University of Rome, Rome, Italy

## 

Despite early surfactant therapy, betterventilator strategies and greater use of noninvasive positive pressure ventilation, bronchopulmonary dysplasia (BPD)continues to be a complication of premature births.

The mainstay of supportive care for infants with severe BPD is mechanical ventilation with an endotracheal tube, however treatmentcan last for a long time and have many complications. When safe extubation is not possible because of multiple failed attempts, tracheostomy is sometimes recommended [1-5]. In all age groups outside the neonatal period, placement of a tracheostomy is considered after a few weeks of mechanicalventilation [6,7]. By contrast, the optimum time and safety procedures have not yet been determined for the placement of a tracheostomy in infants with BPD who need protracted ventilation. Reasons for not performing a tracheostomy in these patientsinclude technical concerns associatedwith small patient size or the need for high ventilator settings. On the other hand the placement of a tracheostomy early in the course of severe BPD could have positive effects such as improved comfort, decreased need for sedation, lower systemic corticosteroid exposure, and enhanced nutrition and growth. 

Recent data [8] suggest that a reasonable approach is that chronically ventilated infants should be assessed at 3 months of age, that is around or shortly after 40 weeks corrected gestational age. If the respiratory support remains high and has been so for 2 months with no evidence of improvement and after multiple attempts to wean the baby off positive pressure ventilation, then infants should be considered for a tracheostomy placement. Another important point highlighted by this report is that tracheostomies should be considered a safe procedure even in infants on high pressures and high concentrations of supplemental oxygen.

Other results [9] suggest a potential association between earlier (<120 days) tracheostomy and better neurodevelopmental outcomes. Actually, while an infant awaits a tracheostomy, the medical focus is often on strategies to allow weaning and limit ventilator-associated lung injury. Following a tracheostomy, the focus may shift to maximizing parent–child interaction and developmental improvement. Furthermore, after tracheostomy, there is often an opportunity to wean the baby off sedating medications, which are frequently associated with increased risk of neurodevelopmental impairment.

In conclusion tracheostomy does not mitigate the significant risk for adverse neurodevelopment that is associated with the many complications of prematurity; however, if tracheostomy is to be performed, earlier surgery may allow opportunities for enhanced neurodevelopmental outcomes.

## References

[B1] Papoff P Cerasaro C Caresta E Barbàra CS Midulla F Moretti C Current strategies for treating infants with severe bronchopulmonary dysplasia JMaternFetal Neonatal Med 2012 25 Suppl 3 15 20 10.3109/14767058.2012.712352 23016612

[B2] Overman AE Liu M Kurachek SC Shreve MR Maynard RC Mammel MC Moore BM Tracheostomy for infants requiring prolonged mechanical ventilation: 10 years’ experience Pediatrics 2013 131 5 e1491 e1496 10.1542/peds.2012-1943 23569088

[B3] Viswanathan S Mathew A Worth A Mhanna MJ Risk factors associated with the need for a tracheostomy in extremely low birth weight infants PediatrPulmonol 2013 48 2 146 150 10.1002/ppul.22599 22644794

[B4] Grover TR Brozanski BS Barry J Zaniletti I Asselin JM Durand DJ Short BL Pallotto EK Dykes F Reber KM Padula MA Evans JR Murthy K High surgical burden for infants with severe chronic lung disease (sCLD) J Pediatr Surg 2014 49 8 1202 1205 10.1016/j.jpedsurg.2014.02.087 25092076

[B5] Murthy K Savani RC Lagatta JM Zaniletti I Wadhawan R Truog W Grover TR Zhang H Asselin JM Durand DJ Short BL Pallotto EK Padula MA Dykes FD Reber KM Evans JR Predicting death or tracheostomy placement in infants with severe bronchopulmonary dysplasia J Perinatol 2014 34 7 543 548 10.1038/jp.2014.35 24651732

[B6] Brook AD Sherman G Malen J Kollef MH Early versus latetracheostomy in patients who require prolonged mechanicalventilation Am J Crit Care 2000 9 5 352 359 10976359

[B7] Carron JD Derkay CS Strope GL Nosonchuk JE Darrow DH Pediatric tracheotomies: changing indications and outcomes Laryngoscope 2000 110 7 1099 1104 10.1097/00005537-200007000-00006 10892677

[B8] Mandy G Malkar M Welty SE Brown R Shepherd E Gardner W Moise A Gest A Tracheostomy placement in infants with bronchopulmonary dysplasia: safety and outcomes Pediatr Pulmonol 2013 48 3 245 249 10.1002/ppul.22572 22570313

[B9] DeMauro SB D'Agostino JA Bann C Bernbaum J Gerdes M Bell EF Carlo WA D'Angio CT Das A Higgins R Hintz SR Laptook AR Natarajan G Nelin L Poindexter BB Sanchez PJ Shankaran S Stoll BJ Truog W Van Meurs KP Vohr B Walsh MC Kirpalani H Eunice Kennedy Shriver National Institute of Child Health and Human Development Neonatal Research Network Developmental outcomes of very preterm infants with tracheostomies J Pediatr 2014 164 6 1303 1310 10.1016/j.jpeds.2013.12.014 24472229 PMC4035374

